# Production technology, nutritional, and microbiological investigation of traditionally fermented mare milk (Chigee) from Xilin Gol in China

**DOI:** 10.1002/fsn3.1298

**Published:** 2019-12-09

**Authors:** Liang Guo, Wei‐Liang Xu, Chun‐Dong Li, Mei Ya, Yuan‐Sheng Guo, Jun‐Ping Qian, Jian‐Jun Zhu

**Affiliations:** ^1^ Xilin Gol Food Testing and Risk Assessment Center Xilingol Vocational College Xilin Gol Institute of Bioengineering Xilinhot China

**Keywords:** Chigee, mare milk, microbiology, nutrition

## Abstract

Mare milk originated from female horses, known as mares, to feed their foals during lactation. The health‐promoting characteristics of traditionally fermented mare milk (Chigee) are well known for the function of clinic treatment in the traditional Mongolian medicine. This study was conducted to investigate the production technology of Chigee and to evaluate the nutritional and microbiological characteristics of mare milk and Chigee based on 188 samples. The nutritional analysis of mare milk and Chigee indicated that lactose significantly decreased from 6.95 ± 0.45% to 2.82 ± 1.65% and acidity and alcoholic content significantly increased to 136.72 ± 57.88°T and 1.22 ± 0.7%, respectively, after spontaneous fermentation of mare milk. The microbiological analysis of Chigee showed that the total lactic acid bacteria (LAB) count varied from 5.32 to 8.56 log cfu/ml and total yeast count varied from 2.41 to 6.98 log cfu/ml. Moreover, the acidity of Chigee rose with the increase in LAB count within limits, and high acidity (≥178°T) inhibited the growth of coliforms. These findings provide an understanding of traditional production technology, nutrition, and microbiology that is fundamental for establishing the food standard of Chigee in China and will contribute to standardize the fermentation process for the industrial production of Chigee in the future.

## Graphical abstract

This study was conducted to investigate the production technology of Chige and to evaluate the nutritional and microbiological characteristics of mare milk and Chige based on large‐scale sampling.

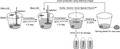

## 1 INTRODUCTION

Milk and milk products are healthy and nutritional foodstuffs consumed by people around the world. Predominantly, ruminant milk (e.g., cow, goat, camel) is utilized to produce milk products, such as yogurt, cheese, cream, and butter. Nowadays, yogurt is economically important and studied by the large number of published articles (Aryana & Olson, 2017). Yogurt possesses a variety of health‐related benefits including angiotensin‐converting enzyme inhibitory activity (Akalin, Unal, & Dinkci, 2018), gut health (Pei, Martin, DiMarco, & Bolling, 2017), and probiotics (Kok & Hutkins, 2018). In addition, yogurt fortified with different components, such as vitamin D, vegetable fiber, whey protein isolates, and grape‐seed oil, exhibits nutritional, microbiological, and functional properties (Mercan, Sert, Karakavuk, & Akın, 2018; Mostafai et al., 2019; Yildiz & Ozcan, 2019; Yildiz‐Akgül, 2018). Compared with yogurt, Chigee, a spontaneously fermented milk product made from mare milk, is popular among the people of Mongolia, Kazakhstan, Kirgizstan, and some regions of Russia and Bulgaria (Danova, Petrov, Pavlov, & Petrova, 2005). Mare milk is a milk secreted by female horses, known as mares, to feed their foals during lactation. Additionally, mare milk, which is not a ruminant milk, is similar to breast milk in nutritional composition (Park, 2009) and can relieve recurrent inflammation (Ellinger, Linscheid, Jahnecke, Goerlich, & Enbergs, 2002). Besides, modified mare milk is a safe substitute for cow milk in infants with allergy (Muraro, Giampietro, & Galli, 2002).

Chigee is extensively consumed by the Mongols of the Xilin Gol region of China. More importantly, it is used as a traditional Mongolian medicine to cure intestinal dyspepsia, hypertension, and dyslipidemia (Rong et al., 2015; Yao et al., 2017). The traditional Mongolian medicine in Xilin Gol established the Chigee therapeutics of anti‐hypertension and anti‐hyperlipidemia. Xilin Gol grassland in China is a natural grazing country and is named “capital of horses” in China. The horse population in Xilin Gol was more than one hundred and fifty thousand in 2019, and mare milk and milk products from this region are notable for their high quality in China. Previous studies conducted nutritional evaluation and microbiological analysis of mare milk from Africa, Mongolia, and Europe (Bornaz et al., 2010; Markiewicz‐Kęszycka et al., 2013; Minjigdorj, Baldorj, & Austbø, 2012). Recent studies have investigated the microbiome of Chigee from Xilin Gol by metagenomic analysis (Gesudu et al., 2016; Guo et al., 2019; Yao et al., 2017). However, Chigee has not been studied well in the fields of traditional production technology, nutrition, and microbiology. In this study, we investigated the artisanal production technology and analyzed nutritional and microbiological properties of mare milk and Chigee based on large‐scale sampling. The findings of this research can be exploited by administrative institutions and industries for the establishment of product standard and commercial production of Chigee, respectively.

## 2 MATERIALS AND METHODS

### 2.1 Collection of samples

**Figure 1:**
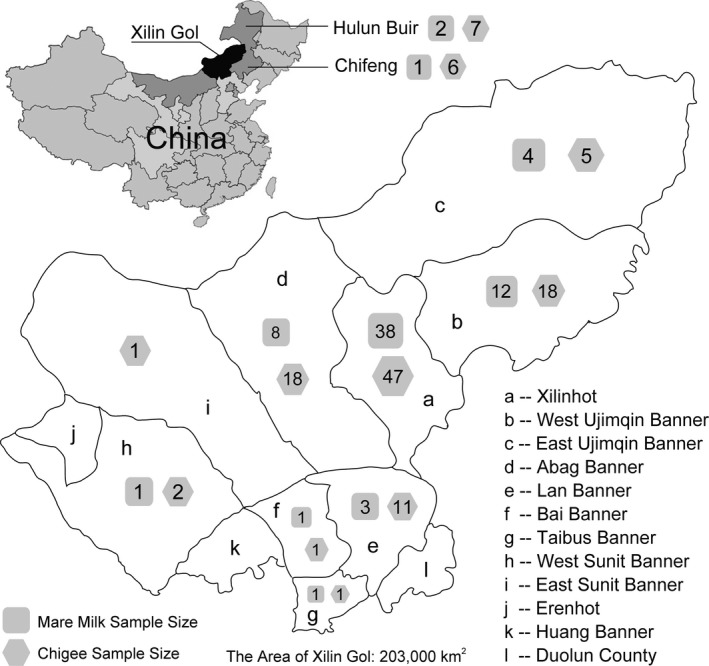
Map showing the administrative divisions of Xilin Gol, sites from where mare milk and Chigee were collected during horse lactation. The location of Xilin Gol in China is highlighted in black in the upper left‐hand corner of the figure. We collected mare milk and Chigee samples from Xilin Gol (68 and 104, respectively), Hulun Buir (2 and 7, respectively), and Chifeng (1 and 6, respectively). Most samples (172 out of 188) were obtained from the nine administrative divisions of Xilin Gol (geographical names are shown in the bottom right‐hand corner)

Seventy‐one mare milk and 117 Chigee samples were collected from Mongolian nomads in Inner Mongolia during horse lactation (June to September). Each sample originated from a herd of mares fed by a nomadic family. Most of the samples (172 out of 188) were obtained from the nine administrative divisions of Xilin Gol; nine samples were obtained from Hulun Buir and seven from Chifeng (Figure 1). A total of 14, 26, 30, and 85 samples were collected from Lan Banner, Abag Banner, West Ujimqin Banner, and Xilinhot, respectively, and 1, 2, 2, 3, and 9 samples were collected from East Sunit Banner, Taibus Banner, Bai Banner, West Sunit Banner, and East Ujimqin Banner, respectively (Figure 1). Samples were collected from almost all areas of the grassland in Inner Mongolia. These dairy samples (500 ml) were collected promptly in sterile self‐sealing bags, placed on ice, and stored at −80°C for future analysis.

### 2.2 Measurement of lactose content, acidity, alcohol content, and pH of mare milk and Chigee

The lactose (China National Food Safety Standard, 2010) content, acidity (China National Food Safety Standard, 2016a), and alcohol (China National Food Safety Standard, 2016b) content were measured according to the protocols in these relevant Chinese national food safety standards. The lactose content was determined by the titration method using Feline's solution (Sinopharm) and methylene blue (Sinopharm). The acidity was determined by the titration method using sodium hydroxide (0.1 mol/L) and phenolphthalein indicator (Sinopharm). The pH of mare milk and Chigee were determined by a pH meter (Mettler Toledo).

### 2.3 Measurement of protein, fat, ash, and minerals contents of mare milk and Chigee

The protein content was determined using the Kjeldahl method (China National Food Safety Standard, 2016c). The fat content was measured using the Soxhlet extraction method (China National Food Safety Standard, 2016d). The ash content was estimated after incineration in an electric muffle furnace (Taisite) at 550°C for 4 hr. The concentrations of calcium, potassium, sodium, magnesium, zinc, iron, copper, and manganese in mare milk and Chigee were determined by atomic absorption spectrophotometry (Thermo Scientific). The concentration of phosphorus was determined by visible spectrophotometry (PerkinElmer).

### 2.4 Microbiological analysis of Chigee

Lactic acid bacteria (LAB) (China National Food Safety Standard, 2016e) was incubated using Man Rogosa Sharpe (MRS) for 72 hr at 36°C according to National Food Safety Standards in China, and the enumeration of LAB was done by counting colonies. The enumeration of yeast (China National Food Safety Standard, 2016f) was calculated using Rose Bengal Agar with chloramphenicol for 5 days at 28°C. The enumeration of coliforms was determined using Violet Red Bile Agar (VRBA) and Brilliant Green Lactose Bile (BGLB) for 48 hr at 36°C (China National Food Safety Standard, 2016g). *Salmonella *spp. (China National Food Safety Standard, 2016h) and *Staphylococcus aureus* (China National Food Safety Standard, 2016i) were detected according to the protocols in these relevant China National Food Safety Standards.

### 2.5 Statistical analysis

The physicochemical data of fresh mare milk and Chigee were using independence‐samples *t* test to determine significant difference. The level of significance was *p* < .01. The data were analyzed using SPSS version 13.0.

## 3 RESULTS AND DISCUSSION

### 3.1 Production technology of naturally fermented Chigee

**Figure 2:**
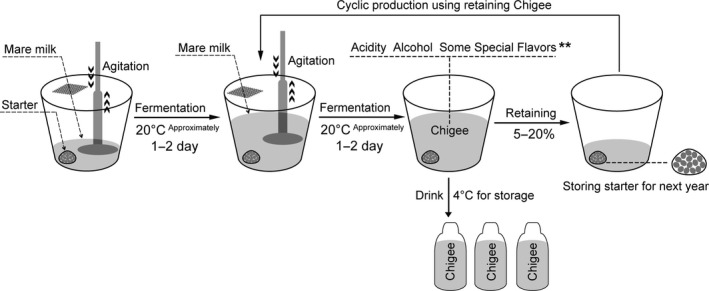
Flowchart and model diagram illustrating the traditional production of Chigee by spontaneous fermentation of mare milk in Xilin Gol. **indicates that the exact duration of spontaneous fermentation depends on acidity, alcohol content, and some special flavors

Chigee is produced by spontaneous fermentation of mare milk. This artisanal production technology was investigated in the nomadic yurts by field survey. Although the different nomadic family had distinctive features in the artisanal technology, the core technology contained the common critical control points. A flow chart model illustrating the production technology of Chigee in Xilin Gol is given in Figure 2. Homemade palm‐sized cloth bag (Hurunge in the Mongolian language) with microbiota from last year's Chigee served as the fermentation starter culture. Mongolian nomads stored homemade starter culture with microbiota from last year to ferment mare milk during horse lactation (June to September). Fresh mare milk was naturally cooled and filtered by gauze. Filtered and cooled mare milk was mixed with starter culture in a porcelain barrel and allowed to spontaneously ferment at ambient temperature (approximately 20°C). Chigee was produced after incubation at ambient temperature (approximately 20°C) for 1–2 days, beaten, and stirred with a wooden stick (100–1,000 times per hour; a maximum of ten thousand per day) to remove carbon dioxide, ensure homogeneity, speed up the process, and eliminate the propagation of other detrimental microbes (e.g., coliforms). Normally, nomads decide the end of fermentation for consumption or sale based on personal favorite and market demand (strong, moderate, and light Chigee), Chigee was bottled in huge quantities (>50% volume) and stored at 4°C to extend shelf life. Chigee with good quality is homogenous, no layers in it, and has fermentative fragrance with sparkling feeling derived from carbon dioxide. Strong Chigee underwent relatively longer spontaneous fermentation with higher acidity and more alcohol as well as carbon dioxide. A small volume of Chigee (5%–20%) from this was retained to serve as a starter culture for the next‐day production. This cycle of Chigee fermentation continued throughout the entire horse lactation period. Homemade starter culture was prepared for next‐year fermentation according to the following steps. Some boiled millets and small fruits were added to the palm‐sized cloth bag, tied with a rope, immersed in the Chigee liquid for several days, and stored at 4°C after drying. The role of millets and small fruits in the preparation of the starter culture was a medium for adsorbing Chigee for adhering microbiota. The millet was unmalted and boiled for enhancing the adsorbing ability of millet. The artisanal production technology was investigated and documented in this study, which could contribute to protect and develop this precious Mongolian cultural heritage.

### 3.2 Nutritional compositions of mare milk and Chigee

**Figure 3:**
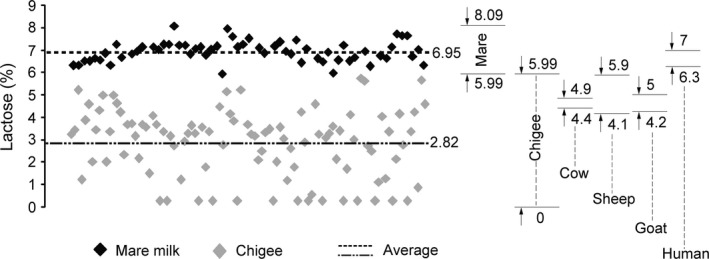
Lactose contents of mare milk (6.95 ± 0.45%; *n* = 60) and Chigee (2.82 ± 1.65%; *n* = 98). The lactose contents of cow, sheep, goat, and human milk were derived from Potočnik et al. (2011)

The change in lactose content after spontaneous fermentation of mare milk is given in Figure 3. Lactose content of fresh mare milk was 6.95 ± 0.45% (range: 5.99%–8.09%; *n* = 60). After spontaneous fermentation, the lactose content of Chigee was 2.82 ± 1.65% (0%–5.99%; *n* = 98). The lactose content in mare milk decreased after spontaneous fermentation. Statistical analysis showed extremely significant difference (*p* < .01) in lactose content between mare milk and Chigee.

We compared mare milk with cow, sheep, goat, and human milk; lactose content in mare milk was the highest (Potočnik, Gantner, Kuterovac, & Cividini, 2011). High lactose content of mare milk (5.99%–8.09%) compared with cow (4.4%–4.9%), sheep (4.1%–5.9%), goat (4.2%–5%), and human (6.3%–7%) milk suggests that more carbon sources could be utilized by LAB and yeasts to produce fermented milk products (e.g., Chigee). We speculated that mare milk with abundant lactose is good for processing into fermented products. Consistent with a previous study (Uniacke‐Lowe, Huppertz, & Fox, 2010), we recorded the lactose content of mare milk similar to that of human milk and more than that of ruminant milk. Additionally, the lactose content of Mongolia mare milk (5.99%–8.09%) is more than that of Andalusian horse (5.41%–6.58%), Standardbred horse (6.08%–7.44%), and Arabian horse (5.57%–6.13%) milk (Park, 2009). In addition, we observed that lactose content was above 7% in the 50% mare milk samples of Xilin Gol. We guessed that lactose, a carbon source, may serve as a prebiotic for microorganisms to yield high‐quality Chigee. We recorded 2.82% lactose in Chigee, which is more than that reported for Chigee (2.2%) and kefir (2%) (Zhang & Cheng, 1997). The test did not detect lactose in 14 out of 98 samples of Chigee. Due to the yield of Chigee from artisanal fermentation of mare milk from Mongolian nomads in Inner Mongolia, the core production technology is consistent, but different nomadic family has distinctive features during handmade process. Thus, the slightly differences in artisanal production technology, surrounding temperature and humidity, sanitary condition, and autochthonic microbes result in the differentiation of physicochemical indexes, such as lactose, acidity, and alcohol content.

**Figure 4:**
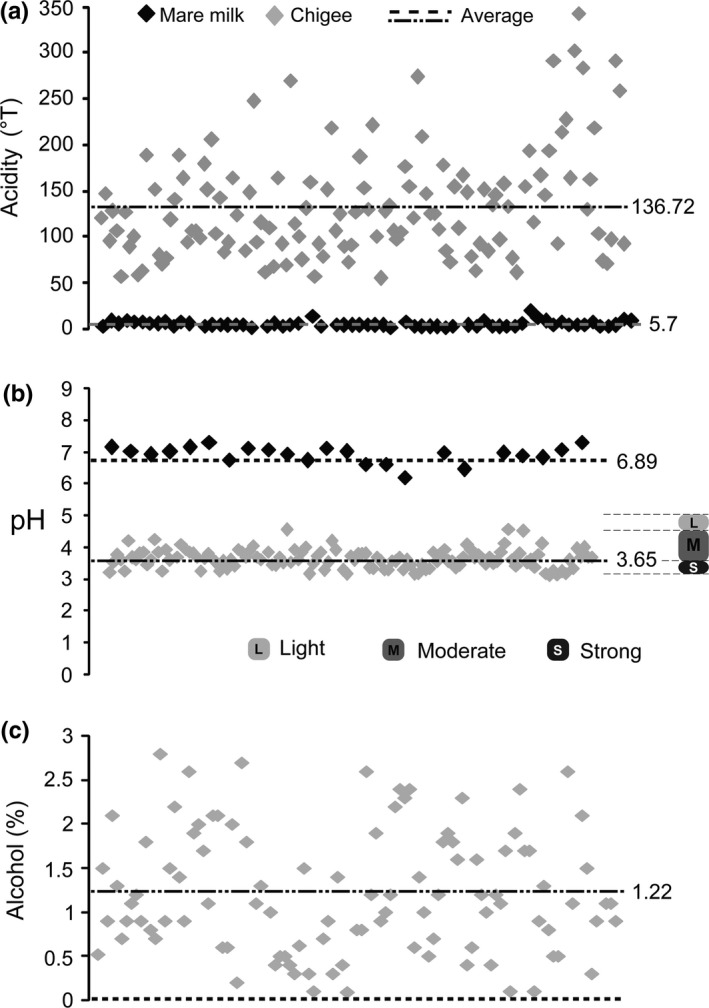
Acidity of mare milk (5.7 ± 3.14°T) and Chigee (136.72 ± 57.88°T) (a). pH of mare milk (6.89 ± 0.2) and Chigee (3.65 ± 0.26) (b). Alcohol content of mare milk and Chigee (1.22 ± 0.7%) (c)

The richness of lactic acid in Chigee was evaluated by acidity using the titration method. In this study, the acidity of mare milk was 5.7 ± 3.14°T (2.13–20.2°T, *n* = 63; Figure 4a). After spontaneous fermentation, the acidity of Chigee reached 136.72 ± 57.88°T (58.7–340°T, *n* = 124; Figure 4a). Statistical analysis showed extremely significant difference in acidity (*p* < .01) between mare milk and Chigee. We observed significant increase in acidity after spontaneous fermentation of mare milk.

Besides, pH value can be applied to evaluate the degree of fermentation of mare milk. The pH of mare milk was 6.89 ± 0.2 (6.35–7.17, *n* = 23; Figure 4b). After spontaneous fermentation, the pH of Chigee was 3.65 ± 0.26 (3.29–4.47, *n* = 126; Figure 4b). Statistical analysis showed extremely significant difference in pH (*p* < .01) between mare milk and Chigee. Chigee is usually classified into three types (strong, moderate, and light) based on pH (Park, 2009). Strong Chigee is a highly acidified product (pH 3.3–3.6), and we obtained 41.3% of strong Chigee from Xilin Gol samples (52 out of 126). Moderate Chigee is a moderately acidified product (pH 3.6–4.5), and we obtained 58.7% of moderate Chigee from Xilin Gol samples (74 out of 126). Chigee in Xilin Gol is known for its fragrance and taste with moderate acidification (Danova et al., 2005). We did not obtain any light Chigee (pH 4.5–5.0) during this process.

Chigee has a unique, slightly sour flavor with a bite from the mild alcoholic content. Fresh mare milk has no alcohol. Nevertheless, the alcohol content of Chigee was 1.22 ± 0.7% (0.09%–2.8%, *n* = 102; Figure 4c). The alcohol content significantly increased after spontaneous fermentation of mare milk due to the function of indigenous yeasts. We speculated that the exact flavor varied between different producers due to the difference in alcohol content and lactic acid richness, and the Chigee in Xilin Gol contained more alcohol than kefir made from cow milk (0.8%) (Zhang & Cheng, 1997).

**Figure 5:**
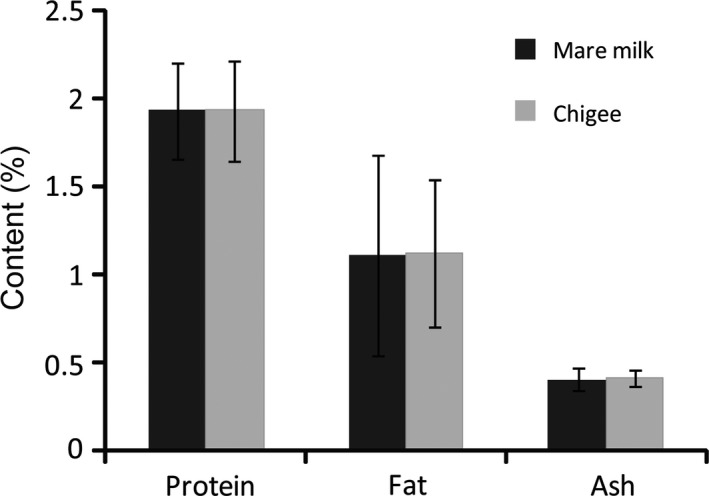
Protein, fat, and ash contents of mare milk and Chigee. There was no significant difference between mare milk and Chigee in protein, fat, and ash contents (*p* > .05)

As shown in Figure 5, the protein content (%) of mare milk was 1.93 ± 0.27 (mean ± *SD*; *n* = 68) and of Chigee was 1.93 ± 0.28 (*n* = 107). The fat content (%) of mare milk was 1.11 ± 0.57 (*n* = 68) and of Chigee was 1.12 ± 0.42 (*n* = 86). The ash content (%) of mare milk was 0.4 ± 0.06 (*n* = 16) and of Chigee was 0.41 ± 0.05 (*n* = 16). Statistical analysis showed no significant difference between mare milk and Chigee in protein, fat, and ash contents (*p* > .05). The above results demonstrated that the traditional fermentation of Chigee did not change the contents of protein, fat, and ash, and consumed lactose by assimilation.

The protein content of mare milk (1.93%) was less than cow (3.4%), sheep (5.6%), goat (3.5%), buffalo (4.7%), camel (3.4%), yak (4.2%), and higher than human milk (0.9%) (Uniacke‐Lowe et al., 2010). The fat content (1.11%) of mare milk was less than that of cow (3.7%), sheep (6.8%), goat (3.8%), buffalo (4.7%), camel (3.8%), yak (5.6%), and human (3.8%) milk (Uniacke‐Lowe et al., 2010). The protein and fat contents of Mongolia horse in Xilin Gol are more than Haflinger and Lusitano (Mariani et al., 2001; Santos & Silvestre, 2008), and less than Arabian and Russian heavy mare (Pieszka & Kulisa, 2003; Stoyanova, Abramova, & Ladoto, 1988).

The ash content in mare milk (0.4%) compared with cow (0.7%), sheep (1%), goat (0.8%), buffalo (0.8%), and human (0.2%) milk (Uniacke‐Lowe et al., 2010) suggests that the mineral content could be less than that of cow, sheep, goat, and buffalo milk and more than that of human milk. There was no difference between mare milk (*n* = 26) and Chigee (*n* = 47) in macroelements and microelements. The concentrations of macroelements in mare milk and Chigee were less than that of other ruminants and more than that of human, and the concentrations of microelements were the lowest in mare milk (Table 1). In addition, the microelement concentrations of mare milk and Chigee were less than that of commercial strawberry‐flavored yogurts and fermented whey beverages (Souza et al., 2019). The results in this study were corresponding to the previous research for the quantity of elements in other mare milk (Park, 2009).

**Table 1:** Macroelements and microelements of mare milk and Chigee

Species	Macroelements (mg/kg)					Microelements (mg/kg)			
	Calcium	Phosphorus	Potassium	Sodium	Magnesium	Zinc	Iron	Copper	Manganese
Mare milk^a^	775 ± 91	603 ± 94	516 ± 130	119 ± 23	57 ± 7	1.86 ± 0.45	0.25 ± 0.15	0.11 ± 0.02	0.018 ± 0.012
Chigee^b^	730 ± 123	593 ± 80	477 ± 122	124 ± 32	56 ± 6	1.81 ± 0.38	0.26 ± 0.16	0.11 ± 0.05	0.019 ± 0.013
Cow^c^	1,220	1,190	1,520	580	120	5.3	0.8	0.6	0.2
Buffalo^d^	1,839	887	1,016	448	190	1.46–7.28	0.42–1.52	0.07–0.21	0.38–0.66
Goat^e^	1,340	1,210	1,810	410	160	5.6	0.7	0.5	0.32
Sheep^f^	1,980	1,300	1,200	500	180	7.5	0.76	0.07	0.007
Human^g^	330	430	550	150	40	3.8	2	0.6	0.07
Mare milk^h^	500–1,300	200–1,200	300–800	167–200	40–110	0.9–6.4	0.22–1.46	0.2–1	0.01–0.05

Note

^a,b^Value represents mean ± *SD*.

^c,d,e,f,g^Adapted from Park (2009).

^h^Adapted from Salimei and Fantuz (2012), value represents the range of content.

### 3.3 Microbiological analysis of Chigee produced from mare milk by spontaneous fermentation in Xilin Gol

Chigee is made from mare milk by spontaneous fermentation of lactose to lactic acid and alcohol, and microbiota plays an essential role in spontaneous fermentation of Chigee. The total number of LAB in the Chigee (*n* = 51) varied from 5.32 to 8.56 log cfu/ml (mean ± *SD*: 7.6 ± 0.68 log cfu/ml). The total number of yeasts in the Chigee (*n* = 65) varied from 2.41 to 6.98 log cfu/ml (mean ± *SD*: 5.56 ± 1.02 log cfu/ml). Our investigation confirmed high microbiological quality of Chigee from Xilin Gol. The pathogens such as *Salmonella *spp. and *Staphylococcus aureus* were not detected in all the samples. However, coliforms were detected in few Chigee samples (*n* = 61). The coliform count varied from 0.3 to 7 log cfu/ml (mean ± *SD*: 3.37 ± 2.06 log cfu/ml).

**Figure 6:**
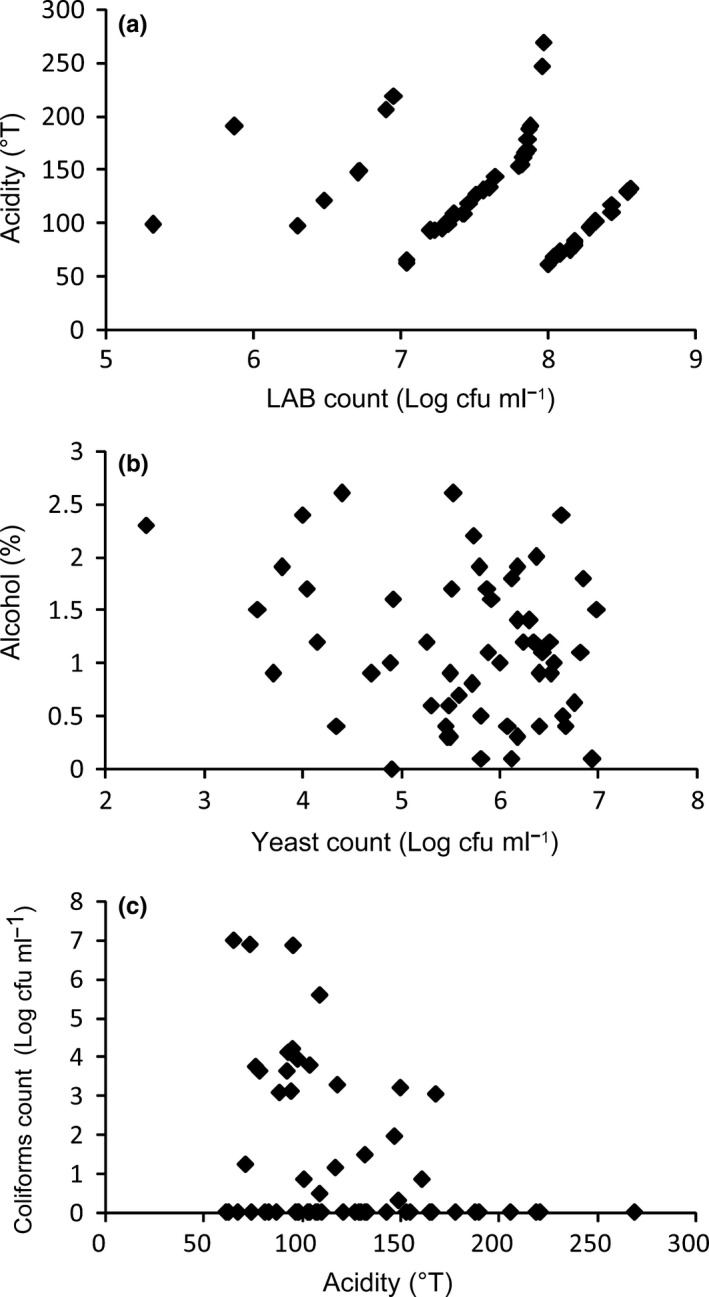
Microbial characteristics of Chigee. Correlation between LAB count and the acidity of Chigee (a), yeast count and the alcohol content of Chigee (b), and coliforms count and the acidity of Chigee (c)

As showed in Figure 6a, the acidity of Chigee rose with increase in LAB within a certain range of LAB count. The correlation between LAB count and acidity suggested that LAB played a major role in the production of lactic acid by fermenting lactose. Chigee was made by fermentation with a mixed microflora, which contained various LAB and yeasts (Gesudu et al., 2016; Guo et al., 2019; Yao et al., 2017). However, the quantity of yeasts in Chigee was not related to the alcohol content (Figure 6b). We speculated that some of yeasts with aerobic respiration could not produce alcohol, and some of LAB could possess alcohol dehydrogenase to oxidize ethanol to acetic acid. In addition, approximately, 41% of the Chigee samples (25 samples) identified to contain coliforms were able to ferment lactose. Coliforms carried out fermentation that resulted in deterioration of milk products (Todaro et al., 2017). Nevertheless, we did not detect coliforms in the Chigee with high acidity (≥178°T) (Figure 6c). We speculated that high acidity may inhibit coliform growth, and LAB and yeasts determined the acidity and alcohol content of Chigee, which restrained the propagation of pathogens, such as coliforms, *Salmonella *spp., and *Staphylococcus aureus*.

Due to the spontaneous fermentation of Chigee by Mongolian nomads in Inner Mongolia, the diversity of homemade starter culture, operational habit of individual, humiture, and hygienic environment of yurts can determine the quality of Chigee such as acidity, alcoholic content, LAB, and yeasts. So, we think that the huge differences in the nutritional and microbiological compositions truly reflect the production technology and natural quality of homemade Chigee of Xilin Gol in the variation.

## 4 CONCLUSIONS

Xilin Gol is a major cradle of Mongolian culture, and Chigee and horses are significant components of this magnificent culture. Chigee is a highly nutritious and health‐promoting traditional dairy food. Nevertheless, the production of Chigee is restricted to the spontaneous fermentation of scarce mare milk in the Mongolian yurt. More importantly, the production technology of Chigee is still not investigated thoroughly and standardized for mass production. In this study, we collected mare milk (*n* = 71) and Chigee (*n* = 117) samples from the herdmen's horses and yurts in Xilin Gol and investigated the traditional technology of Chigee production. The nutritional analysis of mare milk and Chigee suggested that lactose significantly decreased and acidity and alcohol content significantly increased after spontaneous fermentation of mare milk. Nevertheless, there was no change in protein, fat, ash, and mineral contents during the fermentation process. The microbiological analysis revealed the abundance of LAB and yeasts and the absence of *Salmonella *spp. and *Staphylococcus aureus* in Chigee. In addition, the acidity of Chigee rose with the increase in LAB count inhibited the growth of coliforms. The study investigated thoroughly the artisanal production technology of Chigee and its nutritional and microbiological profiles that could contribute to establish the food standard of Chigee in China and to standardize the fermentation technique for the industrial production of Chigee.

## CONFLICT OF INTEREST

All authors declare no conflict of interest.

## ETHICAL STATEMENTS

This study does not involve any human or animal testing.
